# Low recognition of attention deficit hyperactivity disorder in adult patients admitted to the Epilepsy Monitoring Unit

**DOI:** 10.1002/brb3.2731

**Published:** 2022-07-27

**Authors:** Caitlynn Pham, Cayla Roy, Christine Tang, Atul Maheshwari

**Affiliations:** ^1^ Department of Neurology Baylor College of Medicine Houston Texas USA; ^2^ Department of Neuroscience Baylor College of Medicine Houston Texas USA

**Keywords:** ADHD, epilepsy, Epilepsy Monitoring Unit, psychogenic nonepileptic seizures

## Abstract

**Introduction:**

Adult patients with epilepsy (PWE) have an 18% prevalence of comorbid attention deficit hyperactivity disorder (ADHD) compared to a prevalence of 2%–5% in the general population. Recognition of this dual diagnosis is important since stimulant therapy is both safe and effective in this population.

**Methods:**

Here, we aim to determine if PWE have adequate documentation for comorbid ADHD when being admitted to the Epilepsy Monitoring Unit (EMU). A retrospective review was conducted at the Baylor St. Luke's Medical Center EMU for patients presenting between July 2017 and November 2020. Patients were divided into two groups: Group I—patients without a documented ADHD diagnosis or ADHD medications and Group II—patients with a documented ADHD diagnosis and/or taking medications indicated specifically for ADHD.

**Results:**

Of 524 individual patients who presented to the EMU, only 25 patients (4.8%) had documentation of a diagnosis of ADHD and/or ADHD medications (Group II). The proportion of patients in Group II did not significantly differ based on the EMU diagnosis. However, there was a significantly greater number of other psychiatric diagnoses (*p *= .005) and a greater number of psychiatric medications prescribed (*p *< .001) in patients in Group II.

**Conclusion:**

Our study suggests that ADHD is underrecognized and underdiagnosed in patients presenting to the EMU, and screening tools may be useful to help clinicians address seizure comorbidities such as ADHD.

## Graphical Abstract

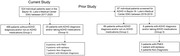

## 1 INTRODUCTION

Adults with epilepsy have been found to have higher rates of comorbid attention deficit/hyperactivity disorder (ADHD) than the general population (Sirven, 2016). Although data in adults are limited compared to data in children with epilepsy, recent studies suggest that ADHD is prevalent in 18% of adults with epilepsy (Ashjazadeh et al., 2019). This prevalence is much higher than expected when compared to a prevalence of 2%–5% of ADHD in the general adult population (Ottman et al., 2011; Polanczyk et al., 2014).

A Diagnostic and Statistical Manual of Mental Disorders (DSM‐V) diagnosis of ADHD requires that the symptoms begin before the age of 12, progress over at least six months, and affect at least two different environments (home, school, work, etc.) (American Psychiatric Association, & American Psychiatric Association, 2013; Rheims & Auvin, 2021). These symptoms progress into adulthood in up to 58% of patients (Barkley et al., 2002). However, a diagnosis may not be made until adulthood in the setting of a well‐compensated disease process in childhood that becomes unveiled in the setting of increased stressors later in life (Solanto, 2019).

It is particularly important to consider the comorbidity of ADHD in adult patients with epilepsy (PWE) because the combination of diagnoses is associated with a decreased quality of life compared to patients with epilepsy alone (Ginsberg et al., 2014). Patients with epilepsy who screen positive for ADHD on the Adult ADHD Self‐Report Scale (ASRS) have also been found to have higher scores of depression and anxiety on the Physicians Health Questionnaire (PHQ‐9) and Generalized Anxiety Disorder Assessment 7 (GAD‐7) scales, respectively, in addition to greater disability, poorer physical health, and lower social functioning (Ettinger et al., 2015). Importantly, therapy with stimulant medication can significantly improve attention deficits with limited concern for seizure exacerbation, validating the need to recognize when patients may have ADHD (Salpekar, 2018).

In this study, we characterized and analyzed the incidence of preadmission ADHD diagnoses in patients who presented to the Baylor St. Luke's Medical Center Epilepsy Monitoring Unit (EMU) between January 2017 and December 2020. Our aim was to identify the degree to which ADHD is documented in patients with epilepsy and psychogenic nonepileptic seizures (PNES) in the EMU setting.

## 2 MATERIALS AND METHODS

This retrospective study was approved by the Baylor College of Medicine Institutional Review Board (H‐32620). We reviewed all patients who presented to the Baylor St. Luke's Medical Center EMU in Houston, Texas, between July 2017 and November 2020. Patient information was extracted from the medical health record (Epic Systems Corporation), including demographic information, admission history, and physical, imaging, and EMU reports. A total of 524 patients who presented during this time were reviewed, ranging between the ages of 17 and 80 upon admission. All patients were evaluated by residents and faculty at Baylor College of Medicine using a standard template. No patients were excluded from this study.

Patients were placed into two groups: Group I—patients without an ADHD diagnosis and not on ADHD medications, Group II—patients with a formal ADHD diagnosis and/or taking medications indicated specifically for ADHD. ADHD medications included generic and brand name derivations of methylphenidate, amphetamine, dextroamphetamine, lisdexamfetamine, and atomoxetine. For medications that may have served a purpose outside of ADHD, a review of the past medical history was performed to ensure that there were no other indications for its use. Factors such as age on admission, sex, handedness, magnetic resonance imaging (MRI) results, EMU assessment and diagnosis, lateralization, seizure/psychiatric medications, other psychiatric diagnoses, and seizure frequency per month were recorded. Handedness, psychiatric diagnoses, current outpatient seizure/psychiatric medications, and seizure frequency per month were reviewed in the medical record. Seizure frequency per month was recorded as an average if the patient presented with a range. EMU assessments and diagnoses were made based on the following: patient histories, MRI, and video electroencephalography results (Maganti & Rutecki, 2013). EMU diagnoses without conclusive evidence of epilepsy were labeled nondiagnostic. Patients with more than one diagnostic category (e.g., epilepsy and PNES) were labeled as “multiple diagnoses”.

A subset of the patients in this retrospective study participated in a prior prospective study screening for ADHD using the Adult ASRS, version 1.1 (Kessler et al., 2007). After the admission history and physical exam were performed, all of these patients were specifically asked if they had a current or prior diagnosis of ADHD and/or took first‐line ADHD medication. The concordance between documentation of ADHD on admission, screening positive for ADHD, and report of an ADHD diagnosis with targeted inquiry in this subset of patients was also evaluated. Mann–Whitney *U* tests were utilized for inferential statistical comparisons. *χ* ^2^ and Fisher's exact tests were conducted to compare frequency distributions. Statistical significance was set at *p *< .05. All statistical tests were performed in GraphPad Prism (v9.2).

## 3 RESULTS

**FIGURE 1:**
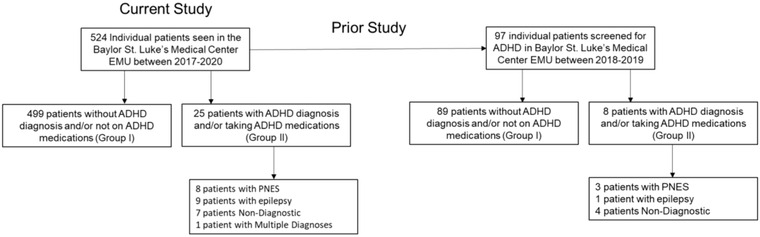
Participant flow. Abbreviations: EMU, Epilepsy Monitoring Unit; GGE = genetic generalized epilepsy; PNES, psychogenic nonepileptic seizures; TLE, temporal lobe epilepsy; PLE, parietal lobe epilepsy

A total of 524 individual patients presented to the Baylor St. Luke's Medical Center Epilepsy Monitoring Unit between January 2017 and December 2020. Of this group, 25 were found to have a documented diagnosis of ADHD and/or taking medications indicated for ADHD on admission (Figure 1). All patients were evaluated and given one of the following diagnoses: psychogenic nonepileptic seizures (PNES), temporal lobe epilepsy (TLE), frontal lobe epilepsy (FLE), parietal lobe epilepsy (PLE), occipital lobe epilepsy (OLE), genetic generalized epilepsy (GGE), physiologic events (PE), nondiagnostic (ND), insular epilepsy (ILE), other focal epilepsy (OFE), and multifocal epilepsy (MFE).

Those with TLE, FLE, PLE, OLE, GGE, ILE, OFE, and MFE are reported in Table 1 under the general term “Epilepsy.” For handedness, 151 patients were not reported due to insufficient data. A positive psychiatric history indicated the presence of anxiety, depression, bipolar disorder, schizoaffective disorder, behavior disorder, obsessive compulsive disorder , posttraumatic stress disorder, panic disorder, history of panic attacks, conversion disorder, borderline personality disorder, and/or mood disorder.

**TABLE 1:** Patient characteristics

		Group I: ADHD and/or ADHD Medication –		Group II: ADHD and/or ADHD medication +			
		*n*	%	*n*	%	Total *n*	*p*‐Value
All patients		499	95.2	25	4.8	524	
Gender	Male	161	32.3	10	40.0	171	.425
	Female	337	67.5	15	60.0	352	
Handedness	Right	301	60.3	17	68.0	318	.220
	Left	45	9.0	0	0	45	
	Both	9	1.8	0	0	9	
EMU diagnosis	PNES	91	18.2	8	32.0	88	.359
	Epilepsy	247	49.5	9	36.0	266	
	PE	19	3.8	0	0	19	
	ND	128	25.7	7	28.0	135	
	Multiple	14	2.8	1	4.0	16	
EEG lateralization of seizure onset	Right	76	15.8	2	8.0	78	.989
	Left	87	17.4	2	8.0	89	
	Bilateral	44	8.8	1	4.0	45	
Other psychiatric history	Present	202	40.5	17	68.0	229	.005**
	Absent	297	59.5	8	32.0	318	

Abbreviations: ADHD, attention deficit hyperactivity disorder; EMU, Epilepsy Monitoring Unit; EEG, electroencephalography; Multiple, multiple diagnoses; ND, nondiagnostic; PNES, psychogenic nonepileptic seizures; PE, physiologic events.

** *p* < .01.

A total of 95.2% of patients were categorized into Group I (no documentation of ADHD) compared to 4.8% in Group II (documentation of comorbid ADHD present). In Group II, eight patients were found to have PNES, nine with epilepsy, seven ND, and one with multiple diagnoses. A greater proportion of PNES patients were in Group II than epilepsy patients, but this was not statistically significant (32.0% vs. 18.2%, respectively, Table 1). However, Group II had a greater proportion of patients with other concurrent psychiatric diagnoses (*p *= .005, Table 1). Finally, PWE and a concurrent ADHD diagnosis and/or ADHD medications also displayed younger age on admission and a greater number of current prescribed psychiatric medications compared to patients without ADHD (*p *< .001, Table 2). The clinical features of the 25 patients who were documented to have a diagnosis of ADHD are detailed in Table 3.

**TABLE 2:** Comparison of continuous variables between groups

	Group I: No comorbid ADHD		Group II: Comorbid ADHD		
	Median	Range	Median	Range	*p*‐Value
Age on admission	37	17–83	22	18–60	<.001
Number of current seizure medications	2	0–6	2	0–4	.984
Number of current other psychiatric diagnoses	0	0–3	1	0–3	.010
Number of current psychiatric medications	0	0–8	1	0–7	<.001
Seizure frequency (per month)	6	0.02–300	30	0.5–107	.371

**TABLE 3:** Detailed characteristics of Group II patients

ID	Age	Sex	Handedness	MRI results	EMU diagnosis	Seizure medications	Other psychiatric diagnoses	Seizure frequency (per month)
1	41	F	Unknown	Pansinus mucosal inflammatory disease	PNES	LTG, LEV, CZP	Anxiety, Depression	83.7
2	19	F	Unknown	Unavailable	ND	GBP, OXC, ZNS	Bipolar disorder, behavior disorder	1
3	60	F	R	Unremarkable	PNES	TPM, PGB, LTG	Depression	82
4	20	F	R	Unavailable	GGE	LEV, ZNS, TPM	None	Unknown
5	21	M	Unknown	Unavailable	ND	LTG, LEV, VPA	None	Unknown
6	56	F	R	Post‐right temporal lobectomy	TLE	LEV	None	270
7	29	F	R	Multifocal intracranial hemosiderin deposits	ND	LEV, ZNS, PHT	Anxiety	3
8	60	F	R	Nonspecific white matter abnormalities	ND	None	Depression	Unknown
9	44	M	R	Post‐right temporal lobectomy	PNES	LEV, VPA, LCM	None	Unknown
10	18	M	Unknown	Unavailable	ND	None	None	Variable
11	22	M	R	Unremarkable	GGE/PNES	CBZ	None	Variable
12	18	F	Unknown	Unremarkable	PNES	PHT, LEV	None	107
13	21	F	R	Unremarkable	TLE	LTG, OXC	None	0.7
14	20	M	R	Unremarkable	GGE	LCM, LEV, CZP	Anxiety	30
15	22	M	R	Unremarkable	TLE	LEV, OXC	Anxiety, depression	2.66
16	24	F	R	Unremarkable	ND	OXC, GBP, LCM	Anxiety, depression	1.33
17	37	M	Unknown	Unavailable	GEN	VPA	Anxiety, depression	2
18	25	F	R	Unavailable	PNES	LEV	Anxiety, depression	52
19	21	M	R	Unremarkable	ND	None	Anxiety, depression	0.5
20	44	F	R	R A1 occlusion	PNES	CZP	Anxiety, depression	66
21	18	F	Unknown	Unremarkable	GGE	LEV	Depression	Variable
22	21	M	R	Unavailable	OFE	LEV, LTG	Depression	Unknown
23	21	F	Unknown	Multifocal thickening	PLE	LTG, LCM, CZP	Depression	63.5
24	31	F	R	Unremarkable	PNES	TPM	Anxiety, depression	30
25	19	M	R	Unremarkable	PNES	VPA, CZP	Schizoaffective disorder	Variable

*Note*: Current seizure medications included levetiracetam (LEV), oxcarbazepine (OXC), zonisamide (ZNS), lamotrigine (LTG), lacosamide (LCM), topiramate (TPM), carbamazepine (CBZ), phenytoin (PHT), valproic acid (VPA), brivaracetam (BRV), clobazam (CBZ), perampanel (PER), rufinamide (RFM), pregabalin (PGB), gabapentin (GBP), clonazepam (CZP), phenobarbital (PHB), and ethosuximide (ESM)).

Abbreviations: GGE, genetic generalized epilepsy; MRI, magnetic resonance imaging; ND, nondiagnostic; OFE, other focal epilepsy; PNES, psychogenic nonepileptic seizures; PE, physiologic events; PLE = parietal lobe epilepsy; TLE, temporal lobe epilepsy.

To examine whether the documented admission history accurately reflected the patient's diagnosis of ADHD, we cross‐referenced the results from this study with data from a prior study where patients were specifically asked whether they had a current or prior diagnosis of ADHD and screened using the Adult ASRS version 1.1. Of the 25 patients who were found to have documented ADHD in the current retrospective review, eight had participated in the prior study. All eight either endorsed having a diagnosis of ADHD or screened positive with the ASRS v1.1, in concordance with their admission history. In contrast, out of a total of 89 patients who did not report an admission ADHD diagnosis in the current study and who also participated in the prior study, 10 patients (11.2%) in the prior study expressed having had a diagnosis when specifically asked. These results indicate that documentation of ADHD is verified when asked, and many EMU patients with a diagnosis of ADHD can be missed if not specifically asked.

## 4 DISCUSSION/CONCLUSION

Out of the 524 patients in our study, 25 patients (4.8%) had documentation on admission supporting a diagnosis of ADHD in the EMU. Compared to an expected prevalence of 18%, this could indicate an underdiagnosis of ADHD in patients who present in an EMU setting. These findings are validated in a review of 97 of these patients who participated in a prior study from our group, showing that 11.2% of patients without ADHD documented on admission to the EMU expressed that they had been given a diagnosis of ADHD when specifically asked. Altogether, 18 of 97 patients (18.6%) admitted to the EMU in the prior study either had an admission diagnosis of ADHD and/or provided a diagnosis when specifically asked, consistent with the expected ∼18% rate of ADHD in this population (Ashjazadeh et al., 2019). Given that comorbid ADHD in PWE can lead to a lower standard of life across various measures, efforts to screen for ADHD in adults with epilepsy could lead to better recognition and management of this population.

Recent literature has shown that ADHD is not only a common comorbidity with epileptic seizures but also with PNES (Say et al., 2015; Simani et al., 2020). Our prior study found that out of all patients diagnosed with PNES, 63.6% screened positive using the Adult ASRS, compared to 27.8% in patients with ES (Dunbar et al., 2019). Our current study is consistent with this previous work, where PNES patients were found to have an admission diagnosis of ADHD at a greater rate than PWE, although this difference was not statistically significant (Table 1). Therefore, screening for ADHD in an EMU setting may be beneficial for both PWE and patients with PNES.

The low recognition of comorbid ADHD has important implications for overall quality of life. PWE and ADHD have been shown to have a lower quality of life than PWE alone in the following dimensions: PHQ‐9 (depression scale) and GAD‐7 (anxiety scale), inability to work due to disability, poorer physical health, and lower social functioning (Ettinger et al., 2015). Congruent with this previous work, we also found a significantly greater number of comorbid psychiatric diagnoses (*p = *.005, Table 2) and a greater number of psychiatric medications prescribed (*p *< .001, Table 2) in patients with an admission diagnosis of ADHD. In addition, the efficacy of first‐line stimulant medications for ADHD, such as methylphenidate, has been reported to be highly effective without significant evidence for seizure exacerbation; therefore, treatment should not be delayed (Chen et al., 2017; McGough, 2016). Patients may also be effectively treated with behavioral therapy in combination with stimulant drugs or behavioral therapy alone (Williams et al., 2016). Altogether, treatment of ADHD may provide a simple and effective benefit to patients with seizures by improving overall quality of life (Adams et al., 2017).

There are several limitations to our study that should be considered. First, the data may be incomplete due to its retrospective nature, and since physicians were not specifically instructed to screen for or document ADHD on admission, a diagnosis could have been omitted from the patient history. In addition, the study only includes the specific population of patients seen in a single‐center tertiary medical center, which may limit the ability to translate findings to other populations. Finally, the diagnosis of ADHD was limited to patient reports, without validation from a formal diagnosis by a trained provider. However, despite the inclusion of ADHD diagnosis based only on patient reports, the prevalence of comorbid ADHD remained surprisingly low.

Future research can be aimed at providing a more streamlined ability to screen, diagnose, and treat patients with seizures and comorbid ADHD. The Adult ASRS is useful in screening for ADHD in this population (Dunbar et al., 2019). While the ASRS does not formally diagnose a patient with ADHD, it may be used as a tool along with other clinical factors to assess which patients require further formal evaluation for ADHD (Chamberlain et al., 2021). However, there are many factors that can impair attention in patients with epilepsy or PNES, including frequent seizures, medication side effects, and comorbid psychiatric diseases such as depression (Salpekar & Mishra, 2014). Future research is necessary to determine to what degree attention deficits in patients with both epileptic and nonepileptic seizures are due to an independent, underlying diagnosis of ADHD.

## CONFLICT OF INTEREST

The authors declare no conflict of interest.

### PEER REVIEW

The peer review history for this article is available at https://publons.com/publon/10.1002/brb3.2731

## Data Availability

The datasets generated in this study are available from the corresponding author upon reasonable request.
